# 

**Published:** 1887-03

**Authors:** 


### AMERICAN MEDICAL ASSOCIATION.

The Thirty-eighth Annual Session will be held in Chicago, Ill., on Tuesday, Wednesday, Thursday and Friday, June 7, 8, 9 and 10, commencing on Tuesday, at 11 A. M.

"The delegates shall receive their appointment from permanently organized State Medical Societies, and such County and District Medical Societies *as are recognized by representation in their respective State Societies,* and from the Medical Department of the Army and Navy, and the Marine Hospital Service of the United States.

Each State, County and District Medical Society entitled to representation shall have the privilege of sending to the Association one delegate for every ten of its regular resident members, and one for every additional fraction of more than half that number; *provided,* however, that the number of delegates for any particular State, territory, county, city or town shall not exceed the ratio of one in ten of the resident physicians who may have signed the Code of Ethics of the Association."

Secretaries of Medical Societies, as above designated, are earnestly requested to forward, *at once,* lists of their delegates.

A member desiring to read a paper before a Section should forward the paper, or its *title* and *length,* (not to exceed twenty minutes in reading), to the Chairman of the Committee of Arrangements at least one month before the meeting.—*By-Laws.*

